# TP53 inhibitor PFTα increases the sensitivity of arsenic trioxide in TP53 wildtype tumor cells

**DOI:** 10.1002/2211-5463.13366

**Published:** 2022-01-26

**Authors:** Haiwei Wang, Xinrui Wang, Liangpu Xu, Ji Zhang

**Affiliations:** ^1^ Medical Research Center Fujian Maternity and Child Health Hospital Affiliated Hospital of Fujian Medical University Fuzhou China; ^2^ State Key Laboratory for Medical Genomics Shanghai Institute of Hematology Rui‐Jin Hospital Affiliated to Shanghai Jiao Tong University School of Medicine Shanghai China

**Keywords:** arsenic trioxide, TP53, TP53 inhibitor PFTα

## Abstract

Arsenic trioxide (ATO) has been shown to be effective in treating acute promyelocytic leukemia. TP53 mutated/null tumor cells are more sensitive to ATO treatment compared to tumor cells carrying wildtype TP53 gene copies. However, it is unclear whether TP53 inhibitors can increase the sensitivity of TP53 wildtype tumor cells to ATO. Here we show that breast, colon, and lung cancer cell lines with mutated/null TP53 are more sensitive to ATO‐induced cell growth inhibition than cells with wildtype TP53. Moreover, inhibition of TP53 by a TP53 inhibitor, PFTα, increased the ATO sensitivity of TP53 wildtype tumor cells, coincident with ATO‐induced cell growth arrest and cell apoptosis. Furthermore, combined treatment with ATO and PFTα synergistically inhibited tumor growth in mouse xenografts *in vivo*. Through microarray transcriptional analysis, we found that ATO‐regulated genes were associated with TP53 and cell cycle signaling pathways. Cotreatment with PFTα enhanced ATO‐induced dynamic transcriptional changes. Overall, our results provide evidence for using TP53 chemical inhibitors to enhance the ATO‐mediated therapeutic response against TP53 wildtype tumor cells.

## Graphical abstract

Here we found that inhibition of TP53 by PFTα sensitized ATO‐induced cancer cell growth arrest and apoptosis in TP53 wildtype tumor cells. Differentially expressed genes after ATO and PFTα treatments were associated with TP53 and cell cycle signaling pathways. Moreover, cotreatment with PFTα enhanced ATO‐induced dynamic transcriptional changes. Our results provided evidence in support of using TP53 chemical inhibitors to enhance the ATO‐mediated therapeutic response against TP53 wildtype tumor cells.

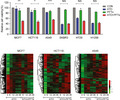

**Abbreviations**
ATO: arsenic trioxide
APL: acute promyelocytic leukemia
RMA: robust multiarray averaging
DAVID: the database for annotation, visualization and integrated discovery
KEGG: Kyoto Encyclopedia of Genes and Genomes
ssGSEA: single sample gene set enrichment analysis

TP53 plays critical roles in tumor development and therapy responses. Half of the tumors are with different types of TP53 mutations and the mutated TP53 could promote tumor growth and metastasis [1, 2]. TP53 wildtype and TP53 mutated/null cancer cells have different mechanisms in responding to cancer treatment, and achieve different clinical outcomes [3]. TP53 stress response systems are required for the efficiency of traditional chemotherapy and radiation therapy [4, 5]. With those treatments, TP53 is activated and induces apoptosis and cell growth arrest through the activation of TP53 target genes [6, 7, 8]. TP53 mutated/null cells usually fail to induce downstream apoptotic genes and are resistant to chemotherapy treatments [9].

Interestingly, reports have suggested that TP53 mutated/null cells are more vulnerable to some other drug insults [10, 11]. For example, the antiglioma drug temozolomide is more effective in TP53 mutated cancer cells than TP53 wildtype cells. And temporary inhibition of TP53 by the chemical inhibitor PFTα could increase the sensitivity of temozolomide in TP53 wildtype cancer cells [12]. Arsenic trioxide (ATO) has been used therapeutically for a thousand years and is very effective in the treatment of acute promyelocytic leukemia (APL) [13, 14, 15]. Cells with defective functions of TP53 are more sensitive to ATO‐induced apoptosis and growth inhibition in multiple myeloma cells [16]. Moreover, ATO could restore the structure of the mutant TP53 and inhibit the growth of cancer cells with structural TP53 mutations [17]. However, whether inhibition of TP53 could increase the sensitivity of ATO in TP53 wildtype tumor cells is unclear.

Here we tested the synergy of the ATO and TP53 inhibitor PFTα in breast, colon, and lung cancer cells with wildtype TP53. We found that the combination of ATO and PFTα could synergistically inhibit tumor growth in TP53 wildtype tumor cells. The TP53 inhibitor PFTα enhanced ATO’s ability to regulate its downstream target genes. Our results suggested a potential therapeutic application of ATO and TP53 inhibitor PFTα in breast, colon, and lung cancer treatment.

## Materials and methods

### Cell lines and cell culture

The human colon carcinoma cell line HCT116, human colon adenocarcinoma cell line HT29, and human lung adenocarcinoma cell line H1299 were cultured in RPMI 1640 supplemented with 10% FBS. The breast cancer cell line SKBR3 was cultured in DMDM‐F12 medium supplemented with 10% FBS. The breast cancer cell line MDA‐MB‐231 was cultured in L15 medium supplemented with 10% FBS. The human non‐small lung cancer cell line A549, colon cancer cell line SW480, SW620, and breast cancer cell line SUM159, BT549 were grown in DMEM supplemented with 10% FBS. All the cell lines were purchased from the Cell Bank/Stem Cell Bank affiliated with the Shanghai Institute of Biochemistry and Cell Biology. All the cells were cultured at 37 °C in a humidified atmosphere with 5% CO_2_.

### Reagents and antibodies

ATO and PFTα of a high analytical grade were purchased from Sigma–Aldrich (St. Louis, MO, USA). Antihuman β‐actin antibody was purchased from Santa Cruz Biotechnology (Santa Cruz, CA, USA). Antihuman PARP and antihuman BCL2, together with all secondary antibodies, were purchased from BD Transduction Laboratories (San Jose, CA, USA).

### Cell viability, cell cycle, and apoptosis analysis

For the cell viability assay, first cells were seeded in 24‐well plates overnight, and then cells were treated with the indicated agent and indicated time course. 100 μL MTT solutions were added to each well for an additional 3 h at 37 °C. The MTT was soluted with 1 ml dimethyl sulfoxide for 1 h and the absorbance was determined and recorded with a Spectra microplate reader DU800 (Beckman Coulter, Brea, CA, USA).

For cell cycle analysis, the trypsinized adherent cells were collected and fixed with 75% ethanol (v/v), stained with propidium iodide, and analyzed using an Aria TM flow cytometer (BD Biosciences Pharmingen, San Diego, CA, USA).

Cytoflow analysis was carried out to determine cell apoptosis. Briefly, cells were seeded in 6‐well plates and exposed to various treatments. The floating and trypsinized adherent cells were then collected and prepared for detection according to the manufacturer’s instructions. Cell apoptosis was detected using FITC‐Annexin V Apoptosis Detection Kit (BD Biosciences Pharmingen, San Diego, CA, USA).

### Subcutaneous model of tumorigenesis

The animal experiments were approved by the Committee on Laboratory Animal Research of Shanghai Jiaotong University, China, and conducted according to the guidelines of the Laboratory Animal Center of Shanghai Jiaotong University School of Medicine. The 6–8‐weeks old female nude mice were purchased from Shanghai Slac Animal Center (Shanghai, China). 10,000 HCT116 cells were injected subcutaneously into the left inguinal area of the mice. After experiments, the mice were sacrificed and the tumors were excised from the body for analysis.

### Western blot analysis

RIPA buffer in the presence of a protease inhibitor cocktail and a phosphorylation inhibitor cocktail were used to extract total protein. Appropriate mount protein was loaded into 10–15% SDS–polyacrylamide gel and transferred onto the nitrocellulose membrane (Millipore, Billerica, MA, USA). Primary antibodies were incubated overnight and secondary antibodies were incubated for 1 h at the appropriate dilutions. The signal was observed and developed with Kodak film by exposure to Enhanced Chemiluminescence plus Western Blotting Detection Reagents (Amersham Biosciences, Piscataway, NJ, USA). Western blot was performed with antibodies against PARP, BCL2, and β‐actin used as a control.

### Microarray hybridization and data mining

Total RNA was amplified and labeled with biotin according to the standard Affymetrix protocol. The fragmented, biotinylated cDNA was hybridized with the Affymetrix Human Genome‐U133 Plus 2.0 array (Affymetrix, Santa Clara, CA, USA). The unprocessed CEL files were Robust Multi‐array Averaging (RMA) normalized in r software (http://www.r‐project.org) using the “affy” library. Raw expression data were annotated with GPL570. The normalized expression data were averaged if multiple probes corresponded to the same gene using the “plyr” library. Differentially expressed genes were selected for the treatment versus no treatment.

### Real‐time PCR

Total RNA was isolated and synthesized to cDNA using Moloney murine leukemia virus reverse transcription kit (Promega, Madison, WI, USA). The expression levels of CCNG2 and SESN2 were detected using 7900HT Fast Real‐Time PCR (Applied Biosystems, Foster City, CA, USA). GAPDH was used as normalization.

### Biological process and Kyoto Encyclopedia of Genes and Genomes (KEGG) signaling pathway analysis

Function enrichment analysis of the KEGG pathway of the ATO plus PFTα‐related genes was carried out using the Database for Annotation, Visualization and Integrated Discovery (DAVID) website (v. 6.8; https://david.ncifcrf.gov) [18, 19]. The Benjamini–Hochberg‐derived step‐up procedure of the false discovery rate was applied to account for multiple hypothesis testing, thus to assess the significance of the biological theme enrichments. The significance threshold was set to *P* < 0.05.

### Single sample Gene Set Enrichment Analysis (ssGSEA)

The relative activity of the TP53 signaling pathway and cell cycle signaling pathway were determined using ssGSEA in the “GSVA” package [20] in r software (v. 4.0, Vienna, Austria).

### Heatmap presentation

Heatmaps were created by the “pheatmap” package using r software. The “pheatmap” package was downloaded from bioconductor. The clustering scale was determined by the “average” method.

### Venn diagram

The Venn diagrams were generated using VENNY 2.1 online for comparing lists.

### Statistical analysis

The boxplots were generated from graphpad Prism 5.0 (San Diego, CA, USA). Statistical analysis was performed using Student’s *t* test and a two‐way ANOVA test. *P* < 0.05 was chosen to be a statistically significant difference. *P < 0.05, **P < 0.01, and ***P < 0.001 are shown.

## Results

### Tumor cells harboring mutated/null TP53 are more sensitive to ATO treatment

**Fig. 1:**
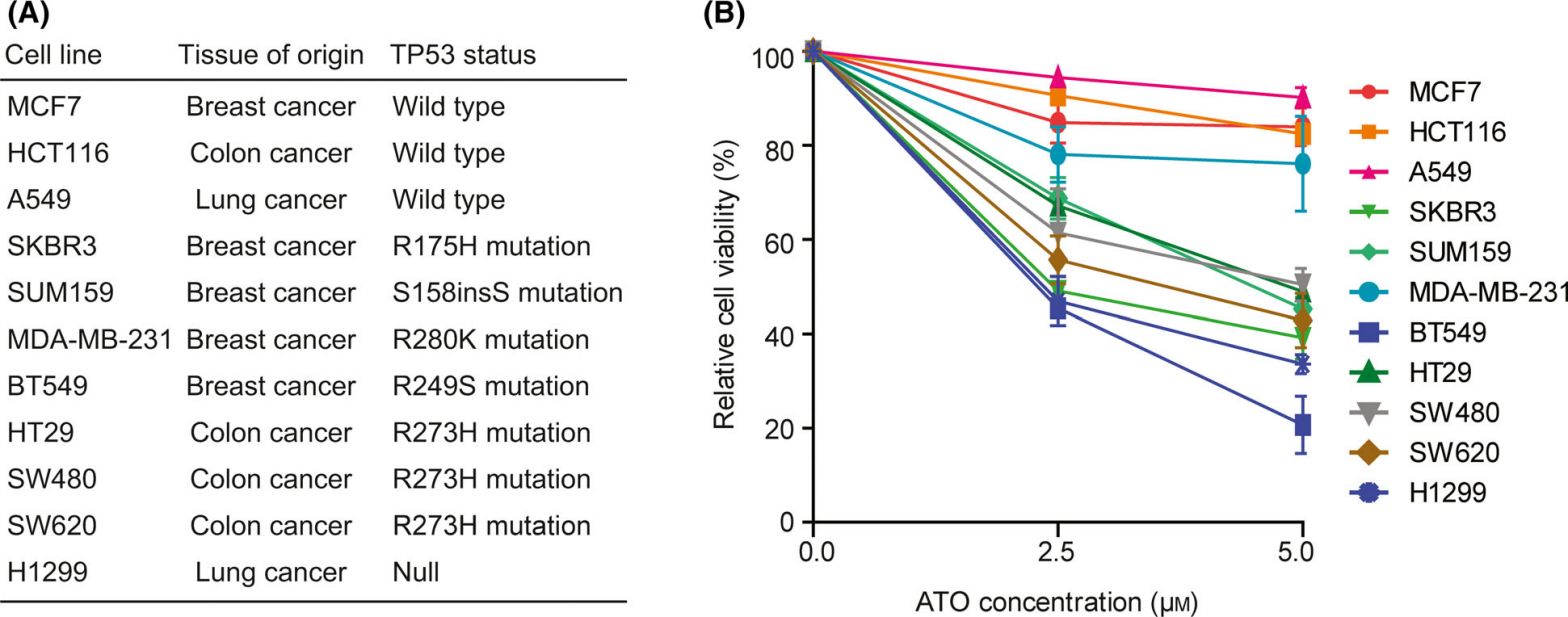
ATO preferentially inhibits TP53 mutated/null cells. (A) Tissue of origin and TP53 status of the cell lines used in our experiments. (B) Cells were treated with 2.5 μm or 5 μm ATO, and the cell viability was tested after 48 h using the MTT assay. Results are means ± SEM from three independent experiments.

Cell lines from breast, colon, and lung cancer patients with wildtype TP53 or different TP53 alterations were used to determine the roles of TP53 in ATO‐induced anticancer activity. A summary of TP53 status and tissue of origin of those cells is shown (Fig. 1A). MCF7, HCT116, and A549 express wildtype TP53, whereas SKBR3, SUM159, MDA‐MB‐231, BT549, HT29, SW480, and SW620 express different mutated TP53. Lung cancer H1299 cells were TP53 null cells.

After 48 h of 2.5 μm or 5 μm ATO treatment, the cell viability was tested through the MTT assay. SKBR3, SUM159, BT549, HT29, SW480, SW620, and H1299 cells with mutated/null TP53 showed great cell growth inhibition; nearly half the percentage of cell viability was inhibited (Fig. 1B). Only MDA‐MB‐231 cells expressed mutant TP53 and seemed not very sensitive to ATO treatment (Fig. 1B). In contrast, HCT116, A549, and MCF7 cell lines harboring the wildtype of TP53 nearly had no growth inhibition after ATO treatment (Fig. 1B). Those results implied that tumor cells harboring mutated/null TP53 were more sensitive to ATO treatment.

### Inhibition of TP53 by PFTα sensitizes ATO‐induced cancer cell growth arrest and apoptosis in TP53 wildtype tumor cells

**Fig. 2:**
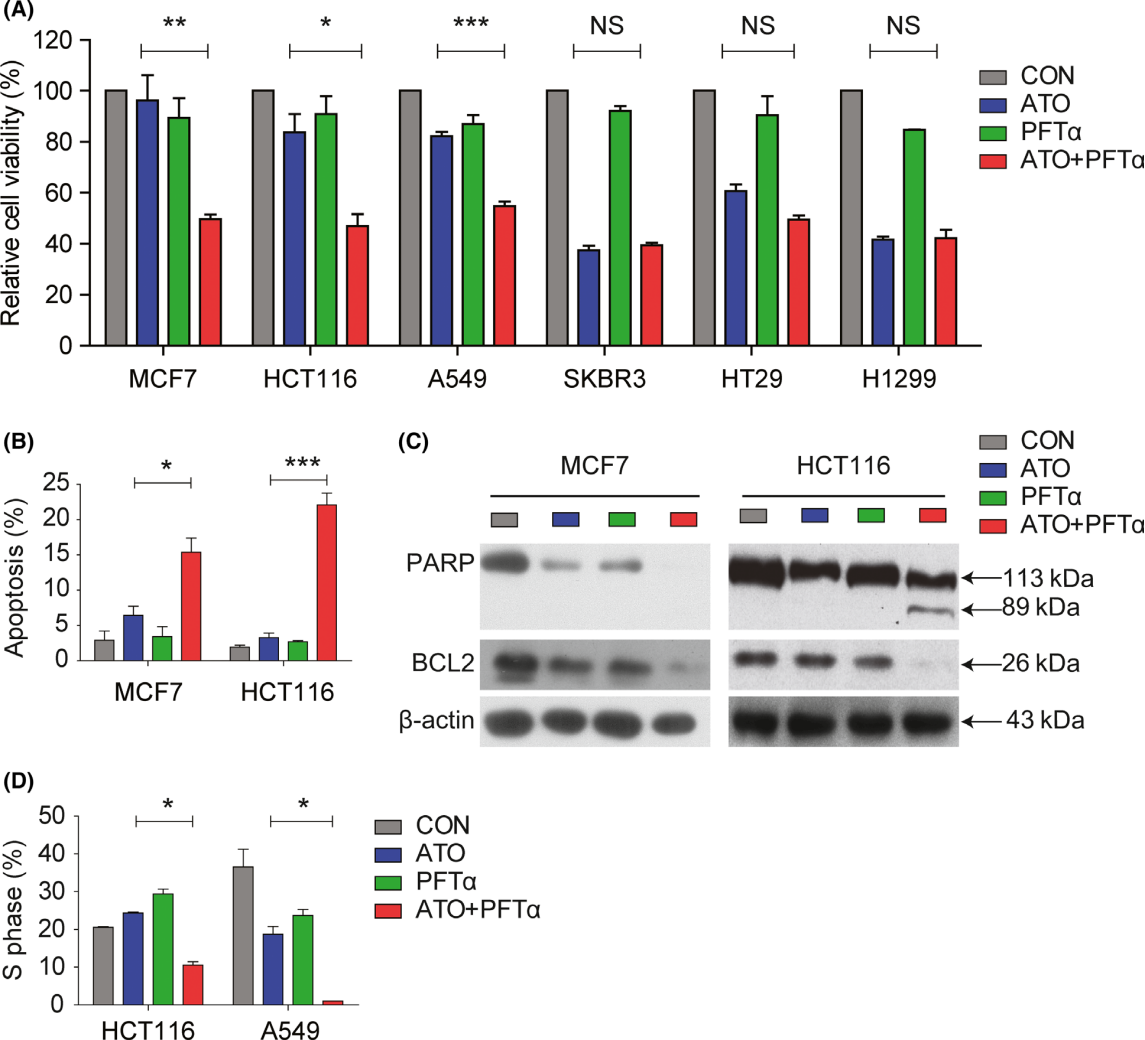
Inhibition of TP53 by PFTα sensitizes ATO‐induced cancer cell growth arrest and apoptosis in TP53 wildtype tumor cells. (A) TP53 wildtype cells MCF7, HCT116, and A549 cells were treated with 5 μm ATO, 20 μm PFTα, or a combination of ATO and PFTα for 48 h. The cell viability was tested. SKBR3, HT29, and H1299 cells were used as negative controls. The error bars indicate means ± SEM from three independent experiments. *P* values were determined using Student’s *t* test. (B) Induction of apoptosis in MCF7 and HCT116 cells under ATO, PFTα, or a combination of ATO and PFTα treatment was evaluated through Annexin V‐FITC and propidium iodide (PI) staining. Data summary and analysis of the apoptotic index represented three independent experiments. The error bars indicate means ± SEM from three independent experiments. *P* values were determined using Student’s *t* test. (C) Induction of apoptosis under ATO, PFTα, or a combination of ATO and PFTα treatment was further evaluated through western blot. PARP and BLC2 expression in MCF7 and HCT116 with the indicated treatments were tested. (D) DNA content in HCT116 and A549 cells after ATO, PFTα, or a combination of ATO and PFTα treatment was determined by PI staining. Percentage of cells in S phase is shown. The error bars indicate means ± SEM from three independent experiments. *P* values were determined using Student’s *t* test.

Since TP53 mutated/null cells were more vulnerable to ATO insult, we wondered if temporary inhibition of TP53 could increase the sensitivity of ATO in TP53 wildtype tumor cells. The TP53 chemical inhibitor PFTα was the first developed TP53 inhibitor that was used to protect from the lethal side effects associated with anticancer treatments by blocking TP53‐dependent transcriptional activation and apoptosis [21]. TP53 wildtype MCF7, HCT116, and A549 cells were treated with ATO 5 μm and/or PFTα 20 μm; cell viability was tested after 48 h. PFTα alone appeared to exert a minor effect on cell growth inhibition, but greatly increased the sensitivity of ATO on HCT116, MCF7, and A549 cells compared with the single ATO treatment (Fig. 2A). Importantly, the synergistic effects of cell growth inhibition were not found in TP53 mutated/null SKBR3, HT29, and H1299 cells (Fig. 2A).

We investigated whether the combination of ATO and PFTα could increase the cell apoptosis in TP53 wildtype tumor cells. The apoptotic rate induced by ATO and PFTα combination treatment was much higher in MCF7 and HCT116 cells than ATO‐alone treatment (Fig. 2B). There was a 6.433 ± 1.309 and 3.280 ± 0.6421 percentage of apoptotic cells in MCF7 and HCT116 after ATO single treatment, but increased to 15.37 ± 2.028 and 22.08 ± 1.682 in MCF7 and HCT116, respectively, after ATO and PFTα combination treatment. Western blot analysis also indicated that PFTα could enhance ATO‐induced apoptosis. Apoptotic biomarkers of suppression of PARP and Bcl‐2 expression were observed in HCT116 and MCF7 cells after combination ATO and PFTα treatment, while no such significant expression changes were tested in the single ATO or PFTα treatment (Fig. 2C). Also, we detected the cleaved PARP in HCT116 after combination ATO and PFTα treatment (Fig. 2C).

The combination of ATO and PFTα on cell cycle progress in TP53 wildtype cells was also studied. HCT116 and A549 cell lines were treated with ATO 5 μm and/or PFTα 20 μm; the DNA content was detected through PI staining. Although a single ATO agent could induce cell arrest in A549 cells, the combination of ATO and PFTα greatly reduced the proportion of S phase cells, from 24.36 ± 0.23 to 10.55 ± 0.93 in HCT116 and from 18.68 ± 2.075 to 0.75 ± 0.25 in A549 cells (Fig. 2D). Those results further confirmed that inhibition of TP53 by PFTα sensitized the ATO therapeutic response by induced cancer cell growth arrest and cell apoptosis.

### ATO and PFTα synergistically inhibit tumor growth *in vivo*

**Fig. 3:**
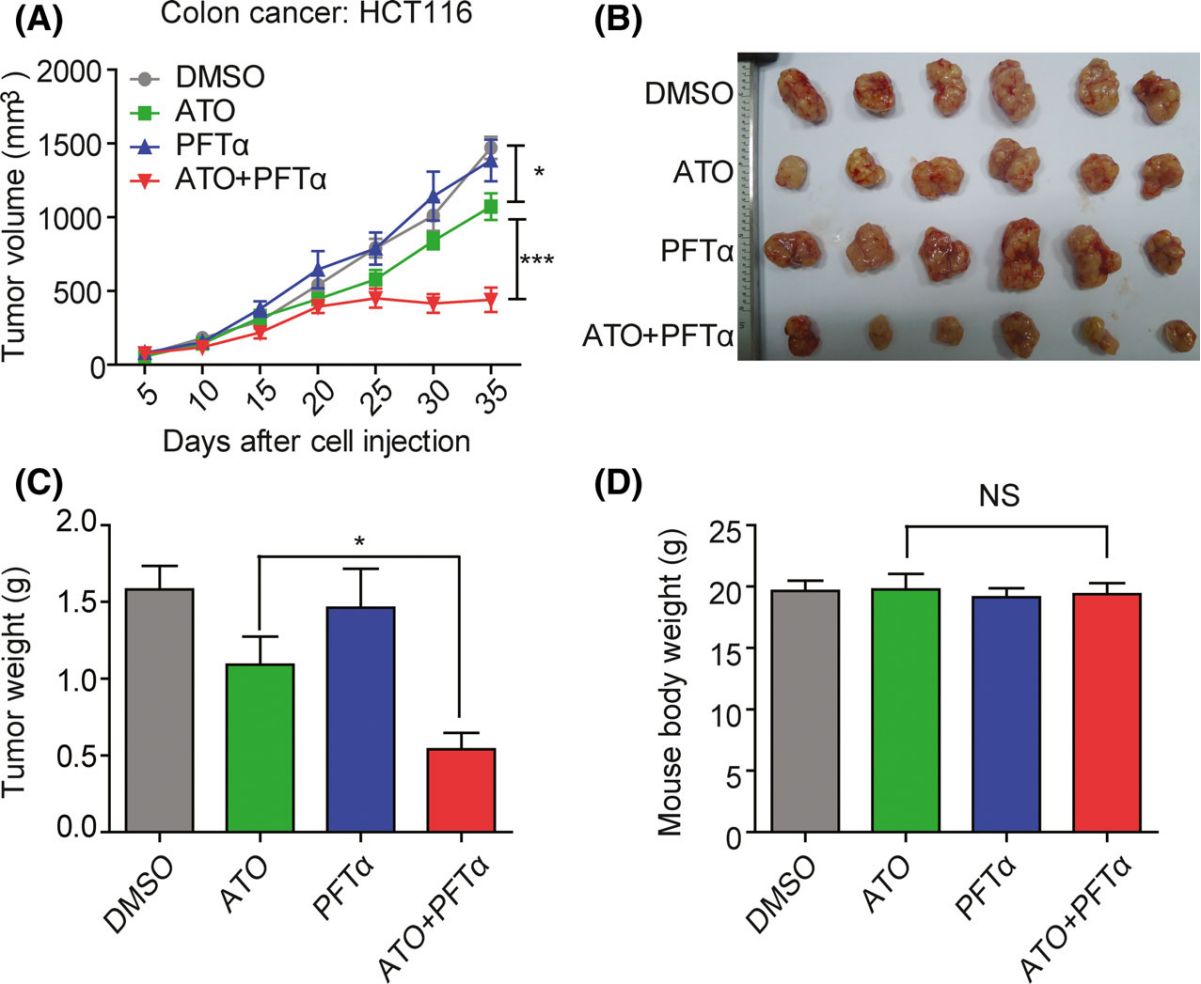
ATO and PFTα synergistically inhibit tumor growth *in vivo*. (A) Tumor growth curve of HCT116 cells with subcutaneous tumors treated with ATO, PFTα, or their combination. The mice (six per group) bearing tumor received intraperitoneal injection 5 mg·kg^−1^·day^−1^ of ATO alone, 2.5 mg·kg^−1^·day^−1^ of PFTα alone, or a combination of ATO and PFTα. Data represent the means ± SEM tumor size of each group. *P* values were determined using a two‐way ANOVA test. (B) Illustration show the tumor excised from each treatment group. (C) The tumor weight of each treatment group is shown. The error bars indicate means ± SEM. *P* values were determined using Student’s *t* test. (D) The mice body weight of each treatment group is shown. The error bars indicate means ± SEM. *P* values were determined using Student’s *t* test.

We also tested whether the combination of ATO and PFTα had the same synergy on tumor growth inhibition *in vivo*. Colon cancer HCT116 cells were subcutaneously inoculated into the immune‐deficient mice. When the xenografts became palpable, animals were treated with 5 mg·kg^−1^ ATO or 2.5 mg·kg^−1^ PFTα or a combination of the two agents for 5 days in 1 week. The tumor size was measured every 5 days. PFTα alone showed no antitumor effect. However, the combination of ATO and PFTα showed significant inhibition of tumor growth than ATO alone (Fig. 3A). The illustrations of excised tumors of each group are shown (Fig. 3B). The average tumor weight in ATO plus PFTα‐treated mice decreased as compared with ATO‐alone treatment (Fig. 3C). Moreover, at this concentration of ATO and PFTα treatment, no significant additional weight loss was observed (Fig. 3D).

### PFTα enhances ATO‐induced dynamic transcriptional changes

**Fig. 4:**
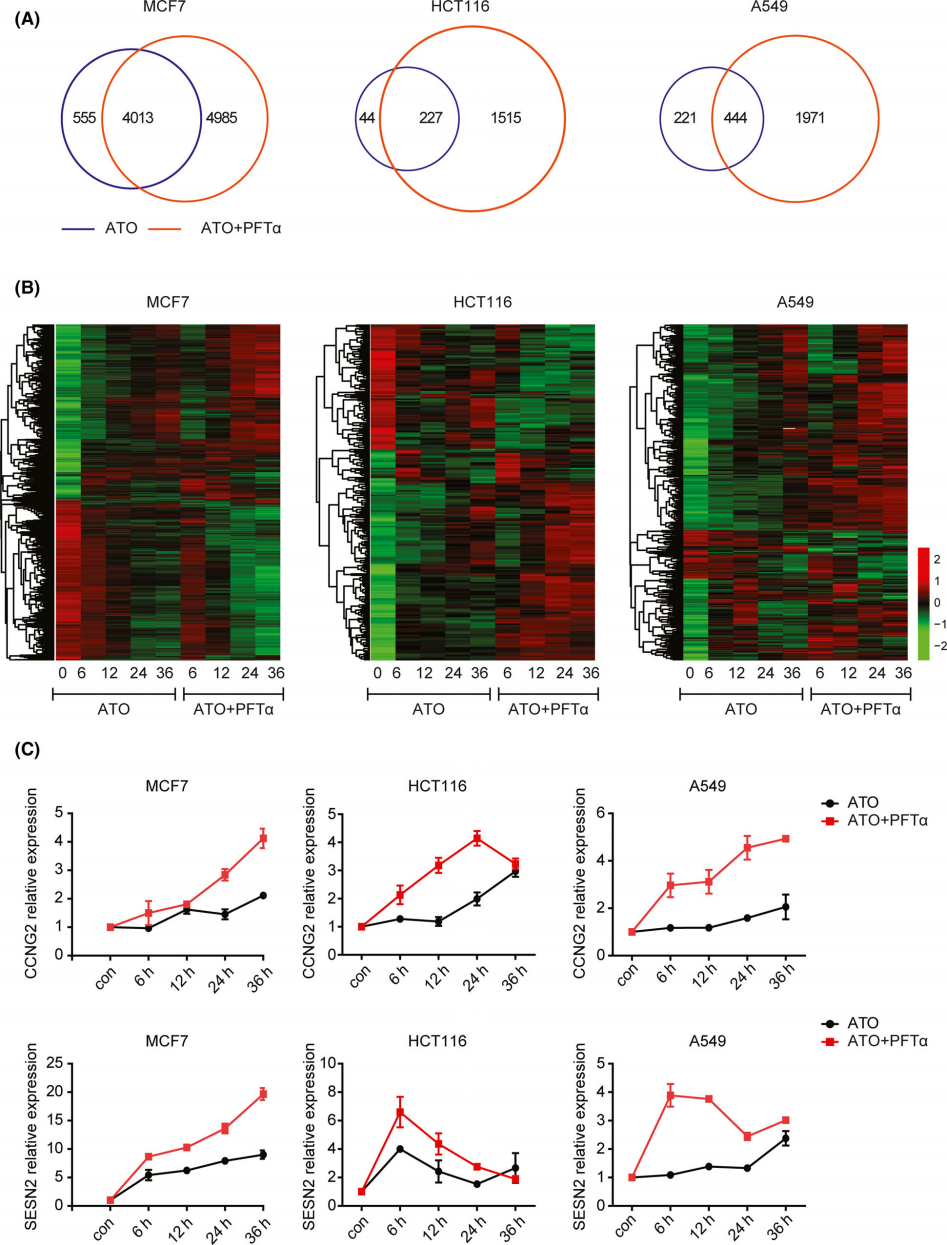
PFTα enhances ATO‐induced dynamic transcriptional changes. (A) Venn diagrams demonstrate the relationship between genes regulated by ATO or combined ATO and PFTα treatment in TP53 wildtype MCF7, HCT116, and A549 cells after ATO treatment at the indicated time. (B) The common regulated genes are further shown through heatmaps. (C) The relative expression levels of *CCNG2* and *SESN2* were tested after ATO or combined ATO and PFTα treatment in TP53 wildtype MCF7, HCT116, and A549 cells at the indicated time. The error bars indicated means ± SEM from three independent experiments.

Next, at the global transcriptional level, we tried to determine the detailed combinational mechanisms of ATO and PFTα in synergistically inducing cell cycle arrest and cell apoptosis. RNA expression profiles from TP53 wildtype MCF7, HCT116, and A549 cells treated with single ATO or the combination of ATO and PFTα at 6 h, 12 h, 24 h, and 36 h were analyzed. Only 122 differentially expressed genes in HCT116 and 200 differentially expressed genes in A549 were identified after ATO treatment (Fig. 4A). The number of ATO‐regulated genes in MCF7 cells was 2050, which was far more than ATO‐regulated genes in HCT116 and A549 cells (Fig. 4A). Furthermore, 4138 genes were modulated by ATO and PFTα treatment in MCF7 cells, 660 genes in HCT116 cells, and 974 genes in A549 cells (Fig. 4A). Overlapping those ATO and PFTα coregulated genes to ATO single‐regulated genes suggested that most of the genes regulated by the ATO single agent were overlapped in ATO plus PFTα‐regulated genes and a great number of genes were only regulated by ATO and PFTα cotreatment (Fig. 4A).

We focused on the common regulated genes between ATO single‐agent treatment and ATO plus PFTα treatment. PFTα greatly enhanced the ability of ATO to regulate its target genes in MCF7, HCT116, and A549 cells (Fig. 4B). In other words, for genes that were up/down‐regulated after ATO‐alone treatment, the combination of PFTα further up/down‐regulated these genes. The expression levels of two TP53 target genes, *CCNG2* and *SESN2*, were further tested using real‐time PCR. ATO is an ROS generator and *SESN2* is known to mediate an antioxidant system to decrease ROS [22]. *CCNG2* is a TP53 target gene regulating cell cycle progress [23, 24]. We showed that the additional PFTα enhanced ATO’s ability to upregulate its downstream target genes *CCNG2* and *SESN2* in MCF7, HCT116, and A549 cells (Fig. 4C).

### ATO plus PFTα‐regulated genes are associated with TP53 and cell cycle signaling pathways

**Fig. 5:**
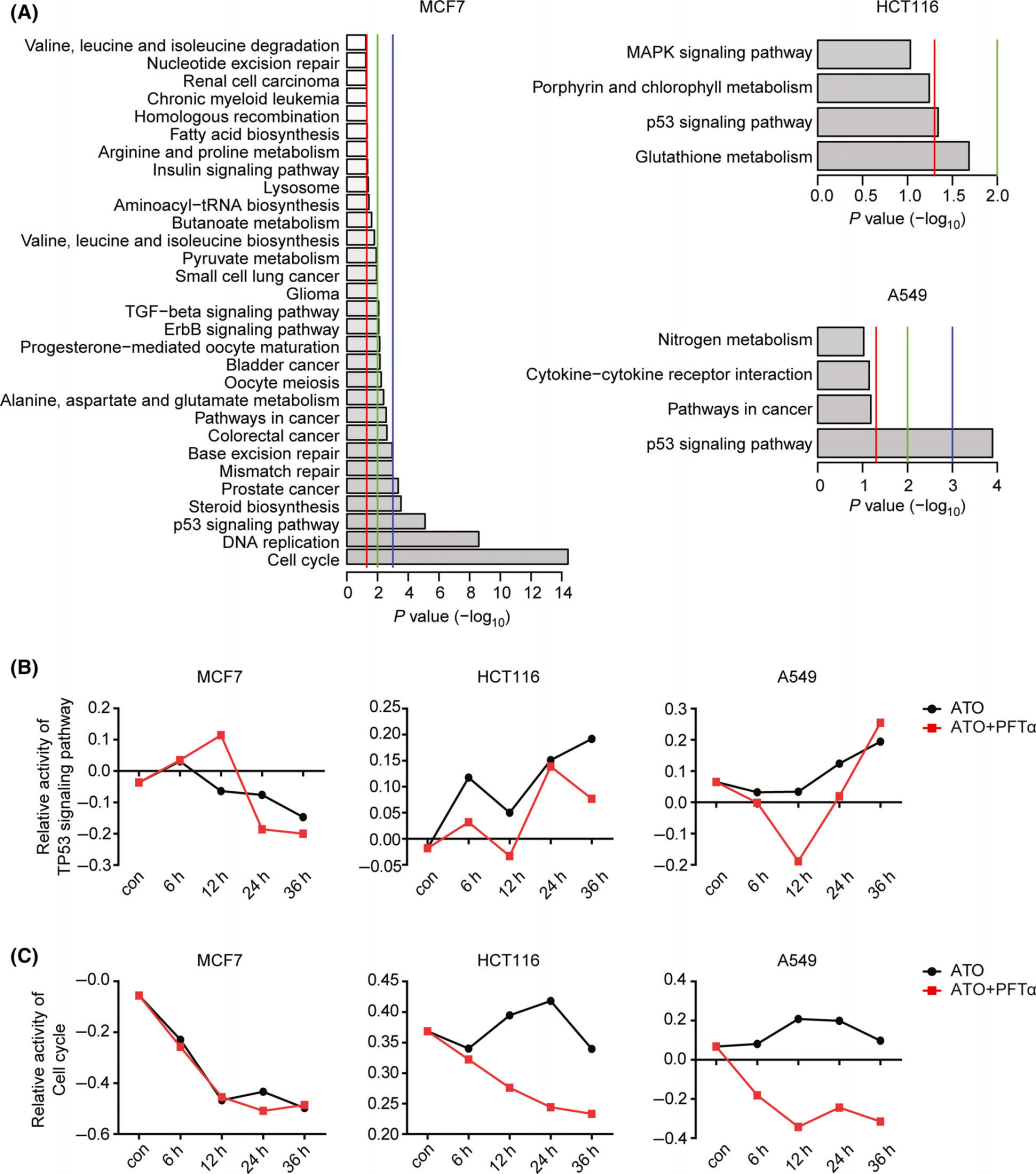
ATO plus PFTα‐regulated genes are associated with TP53 and cell cycle signaling pathways. (A) Functional DAVID enrichment analysis of the pathways associated with ATO plus PFTα‐regulated genes in TP53 wildtype MCF7, HCT116, and A549 cells. The most enriched pathways are shown and the *P* values demonstrated. (B) The relative activity of the TP53 signaling pathway was tested after ATO or combined ATO and PFTα treatment in TP53 wildtype MCF7, HCT116, and A549 cells at the indicated time. (C) The relative activity of the cell cycle signaling pathway was tested after ATO or combined ATO and PFTα treatment in TP53 wildtype MCF7, HCT116, and A549 cells at the indicated time.

To reveal the functional relevance of the common regulated genes between ATO single‐agent treatment and ATO plus PFTα treatment, we performed KEGG signaling pathway enrichment analysis using DAVID. The KEGG TP53 signaling pathway was highly enriched in MCF7 cells, HCT116 cells, and A549 cells (Fig. 5A). Besides the TP53 signaling pathway, some other cellular pathways, like cell cycle, MAPK signaling pathway, and the TGFβ signaling pathway were also associated with ATO functions (Fig. 5A). Reports have shown that the MAPK pathway inhibitor SB203580 [25, 26, 27] sensitized tumor cells to ATO‐induced growth inhibition. Those results showed that ATO was a multitarget drug, and affected multiple signaling pathways.

Moreover, the TP53 signaling pathway activity was determined by single‐sample gene set enrichment analysis (ssGSEA). We found that the relative activity of the TP53 signaling pathway was increased after ATO treatment in HCT116 and A549 tumor cells (Fig. 5B). Moreover, the activation of the TP53 signaling pathway was inhibited by additional PFTα treatment (Fig. 5B). On the contrary, in MCF7 cells the TP53 signaling pathway activity was decreased by ATO or ATO plus PFTα treatment (Fig. 5B).

Previous results showed that the inhibition of TP53 by PFTα sensitized ATO therapeutic response by induced cancer cell growth arrest, and then the relative activity of the cell cycle was tested in MCF7, HCT116, and A549 cells. The single ATO agent did not decrease the relative activity of the cell cycle in HCT116 and A549 (Fig. 5C). However, the relative activity of the cell cycle signaling pathway was significantly decreased by ATO combined PFTα treatment in HCT116 and A549 tumor cells (Fig. 5C). On the contrary, ATO alone could inhibit the relative activity of the cell cycle in MCF7 cells (Fig. 5C).

## Discussion

ATO is very effective in the treatment of APL [13, 14, 15]. With combinations with all‐trans retinoid acid, more than 90% APL patients are cured [28, 29]. The effects of ATO and all‐trans retinoid acid in APL patients are mainly related to the oncogene PML‐RARα [22, 30, 31, 32, 33]. However, the combinations of ATO and all‐trans retinoid acid in solid tumors have not achieved satisfactory clinical outcomes. In solid tumors, ATO regulated the m‐TOR signaling pathway [34], MAPK signaling pathway [25, 26, 27] and Hedgehog signaling pathway [35, 36]. The m‐TOR signaling pathway inhibitor rapamycin [34], MAPK signaling pathway inhibitor SB203580 [25, 26, 27] and Hedgehog signaling pathway inhibitor itraconazole [37] all increased the sensitivity of ATO in solid tumors. Yet more detailed functions of ATO in solid tumor cells should be studied.

The functions of ATO in solid tumor cells are also associated with the TP53 signaling pathway. Indeed, cells with defect functions of TP53 are more sensitive to ATO‐induced apoptosis and growth inhibition in multiple myeloma, breast cancer, lung cancer, or colon cancer cells. The TP53 inhibitor PFTα is used to protect mice from the lethal side effects associated with anticancer treatment by blocking TP53‐dependent transcriptional activation. Although several studies have reported that PFTα has p53‐independent effects in cells [38, 39, 40], our results showed that PFTα increased the sensitivity of ATO in TP53 wildtype tumor cells *in* *vitro* and *in* *vivo*. The additional PFTα treatment could enhance ATO’s ability to regulate its downstream target genes. Our results suggested a potential therapeutic implication of ATO and TP53 inhibitor PFTα in breast, colon, and lung cancer treatment.

However, the use of the small‐molecule inhibition of TP53 in some anticancer therapies have attracted little attention [41]. A great concern about the clinical use of TP53 inhibitors is whether TP53 small‐molecule inhibitors could promote undesirable systemic side effects, including the development of independent cancers in other organs [42]. An observation in this study was that ATO combined TP53 inhibitor treatment was without obvious adverse effects in mice. However, the long‐term adverse effects of ATO and PFTα in clinical usage should be estimated. And the long‐term consequences of this therapeutic approach will need to be investigated in detail.

## Conclusion

The TP53 inhibitor PFTα increases the sensitivity of ATO in TP53 wildtype breast, colon, and lung tumor cells.

## Conflict of interest

The authors declare that they have no conflict of interest.

## Author contributions

HW designed the study and wrote the article. HW did the experiments. XW and LX performed the data analysis. JZ designed the study and supervised the work.

## Data Availability

The transcriptional profiling of MCF7, HCT116, and A549 cells with ATO or ATO combined PFTα treatments generated during the current study are available in GSE124347 repositories.
